# Routine childhood immunisation during the COVID-19 pandemic in Africa: a benefit–risk analysis of health benefits versus excess risk of SARS-CoV-2 infection

**DOI:** 10.1016/S2214-109X(20)30308-9

**Published:** 2020-07-17

**Authors:** Kaja Abbas, Simon R Procter, Kevin van Zandvoort, Andrew Clark, Sebastian Funk, Tewodaj Mengistu, Dan Hogan, Emily Dansereau, Mark Jit, Stefan Flasche, LSHTM CMMID COVID-19 Working Group, Rein M G J Houben, W John Edmunds, Christian Julian Villabona-Arenas, Katherine E Atkins, Gwenan M Knight, Fiona Yueqian Sun, Megan Auzenbergs, Alicia Rosello, Petra Klepac, Joel Hellewell, Timothy W Russell, Damien C Tully, Jon C Emery, Hamish P Gibbs, James D Munday, Billy J Quilty, Charlie Diamond, Carl A B Pearson, Quentin J Leclerc, Emily S Nightingale, Yang Liu, Akira Endo, Arminder K Deol, Adam J Kucharski, Sam Abbott, Christopher I Jarvis, Kathleen O'Reilly, Thibaut Jombart, Amy Gimma, Nikos I Bosse, Kiesha Prem, Stéphane Hué, Nicholas G Davies, Rosalind M Eggo, Samuel Clifford, Graham Medley

**Affiliations:** a London School of Hygiene & Tropical Medicine, London, UK; b Gavi, the Vaccine Alliance, Geneva, Switzerland; c Bill & Melinda Gates Foundation, Seattle, WA, USA; d Public Health England, London, UK; e University of Hong Kong, Hong Kong Special Administrative Region, China

## Abstract

**Background:**

National immunisation programmes globally are at risk of suspension due to the severe health system constraints and physical distancing measures in place to mitigate the ongoing COVID-19 pandemic. We aimed to compare the health benefits of sustaining routine childhood immunisation in Africa with the risk of acquiring severe acute respiratory syndrome coronavirus 2 (SARS-CoV-2) infection through visiting routine vaccination service delivery points.

**Methods:**

We considered a high-impact scenario and a low-impact scenario to approximate the child deaths that could be caused by immunisation coverage reductions during COVID-19 outbreaks. In the high-impact scenario, we used previously reported country-specific child mortality impact estimates of childhood immunisation for diphtheria, tetanus, pertussis, hepatitis B, *Haemophilus influenzae* type b, *Streptococcus pneumoniae*, rotavirus, measles, meningitis A, rubella, and yellow fever to approximate the future deaths averted before 5 years of age by routine childhood vaccination during a 6-month COVID-19 risk period without catch-up campaigns. In the low-impact scenario, we approximated the health benefits of sustaining routine childhood immunisation on only the child deaths averted from measles outbreaks during the COVID-19 risk period. We assumed that contact-reducing interventions flattened the outbreak curve during the COVID-19 risk period, that 60% of the population will have been infected by the end of that period, that children can be infected by either vaccinators or during transport, and that upon child infection the whole household will be infected. Country-specific household age structure estimates and age-dependent infection-fatality rates were applied to calculate the number of deaths attributable to the vaccination clinic visits. We present benefit–risk ratios for routine childhood immunisation, with 95% uncertainty intervals (UIs) from a probabilistic sensitivity analysis.

**Findings:**

In the high-impact scenario, for every one excess COVID-19 death attributable to SARS-CoV-2 infections acquired during routine vaccination clinic visits, 84 (95% UI 14–267) deaths in children could be prevented by sustaining routine childhood immunisation in Africa. The benefit–risk ratio for the vaccinated children is 85 000 (4900–546 000), for their siblings (<20 years) is 75 000 (4400–483 000), for their parents or adult carers (aged 20–60 years) is 769 (148–2700), and for older adults (>60 years) is 96 (14–307). In the low-impact scenario that approximates the health benefits to only the child deaths averted from measles outbreaks, the benefit–risk ratio to the households of vaccinated children is 3 (0–10); if the risk to only the vaccinated children is considered, the benefit–risk ratio is 3000 (182–21 000).

**Interpretation:**

The deaths prevented by sustaining routine childhood immunisation in Africa outweigh the excess risk of COVID-19 deaths associated with vaccination clinic visits, especially for the vaccinated children. Routine childhood immunisation should be sustained in Africa as much as possible, while considering other factors such as logistical constraints, staff shortages, and reallocation of resources during the COVID-19 pandemic.

**Funding:**

Gavi, the Vaccine Alliance; Bill & Melinda Gates Foundation.

## Introduction

Vaccines have substantially improved health and reduced mortality, particularly among children in low-income countries.^1^, ^2^, ^3^ Access to vaccines in these countries accelerated after the formation of Gavi, the Vaccine Alliance, in 2000.^4^ This access needs to be sustained to further advance the public health gains and maintain progress towards goals such as the elimination of polio, measles, rubella, and maternal tetanus.^5^ WHO launched the Immunization Agenda 2030 strategy in 2020 to accelerate progress towards equitable access and use of vaccines over the new decade.^6^ However, ensuring everyone has access to immunisation services has been challenging, with a quarter of children in the Africa region not receiving three doses of diphtheria-tetanus-pertussis (DTP3) in 2018.^7^ An additional challenge is posed by the COVID-19 pandemic,^8^ which has necessitated physical distancing measures to mitigate or delay the epidemic to avoid health-care systems being overwhelmed.^9^, ^10^

**Research in context**

**Evidence before this study**

National immunisation programmes globally are at risk of disruption due to the severe health system constraints caused by the ongoing COVID-19 pandemic and the physical distancing measures to mitigate the outbreak. Outbreaks of vaccine-preventable disease have been observed during previous interruptions to routine immunisation services, such as during the 2013–16 Ebola outbreak in west Africa, when most health resources were shifted towards the Ebola response and decreased vaccination coverage led to consequent outbreaks of measles and other vaccine-preventable diseases. WHO has recommended that countries conduct individual benefit–risk assessments to support decision making on sustaining immunisation services during the COVID-19 pandemic based on the local transmission dynamics of severe acute respiratory syndrome coronavirus 2, the epidemiology of vaccine-preventable diseases, and the characteristics of their immunisation and health systems. To the best of our knowledge, this is the first benefit–risk analysis on sustaining routine childhood immunisation in African countries during the COVID-19 pandemic.

**Added value of this study**

We estimated the benefit–risk ratio by comparing the deaths prevented by sustaining routine childhood immunisation for diphtheria, tetanus, pertussis, hepatitis B, *Haemophilus influenzae* type b, *Streptococcus pneumoniae*, rotavirus, measles, *Neisseria meningitidis* serogroup A, rubella, and yellow fever with the excess COVID-19 deaths associated with vaccination clinic visits. The benefit of routine childhood immunisation programmes in all 54 African countries was found to be greater than the COVID-19 risk associated with these vaccination clinic visits.

**Implications of all the available evidence**

Routine childhood immunisation programmes should be safeguarded for continued service delivery and prioritised for the prevention of infectious diseases, as much as is logistically possible, as part of delivering essential health services during the COVID-19 pandemic in Africa. The current immunisation service models will require adaptation, including physical distancing measures, personal protective equipment, and good hygiene practices for infection control at the vaccination clinics.

The severe acute respiratory syndrome coronavirus 2 (SARS-CoV-2) emerged in December, 2019, when it caused cases of COVID-19 in Wuhan, China.^11^ As of July 10, 2020, there were 12 015 193 confirmed cases and 549 247 confirmed deaths across 216 countries and territories.^8^ All African countries have reported cases of COVID-19, with the majority reporting local transmission and rapidly rising case numbers.^12^ The prevention and control measures to suppress and mitigate the COVID-19 outbreak in Africa during the upcoming months will place immense pressures on the national health systems in their provision of essential health services, including the Expanded Programme on Immunization (EPI) and routine vaccination of infants.^13^

On March 26, 2020, WHO and the Pan American Health Organization issued guidance on the operation of immunisation programmes during the COVID-19 pandemic.^14^, ^15^ The guidance called for temporary suspension of mass vaccination campaigns and a benefit–risk assessment to decide on conducting outbreak response mass vaccination campaigns; routine immunisation programmes were advised to be sustained in places where essential health services had operational capacity of adequate human resources and vaccine supply, while maintaining physical distancing and other infection control measures.

Our aim was to compare the health benefits of sustaining routine childhood immunisation in Africa with the risk of acquiring SARS-CoV-2 infection through visiting routine vaccination service delivery points. Specifically, we did a benefit–risk analysis of vaccine-preventable deaths averted by sustaining routine childhood immunisation in comparison with excess COVID-19 deaths from SARS-CoV-2 infections acquired by visiting routine vaccination service delivery points.

## Methods

### Assumptions

We assessed the benefit and risk of continued routine childhood immunisation during the COVID-19 pandemic in all 54 African countries. We focused on the delivery of infant immunisation at: (1) 6, 10, and 14 weeks of age for diphtheria, tetanus, and pertussis (DTP), hepatitis B, *Haemophilus influenzae* type b, *Streptococcus pneumoniae*, and rotavirus (hereafter called EPI-1); (2) 9 months for measles (MCV1), rubella (RCV1), *Neisseria meningitidis* serogroup A, and yellow fever (hereafter called EPI-2); and (3) 15–18 months for the second dose of measles (MCV2; hereafter called EPI-3). The target age for *N meningitidis* serogroup A routine immunisation varies by country and is given along with the first or second dose of measles vaccine; it is given at 9 months in Central African Republic, Chad, Côte d'Ivoire, Mali, Niger, and Sudan, at 15 months in Burkina Faso, and at 18 months in Ghana.^16^ We did not consider BCG or hepatitis B birth dose because they are recommended for administration shortly after birth and thus were assumed not to require an additional vaccination visit, even though home births or delayed administration might be common in some parts of Africa.

During the period of SARS-CoV-2 circulation, we assumed that contact-reducing measures were in place and that although those measures did not contain the outbreak, they did substantially flatten the epidemic curve. However, with gradual easing of interventions and in the absence of a vaccine, we assumed that SARS-CoV-2 transmission will infect around 60% of the population. In both alternative scenarios (uncontrolled epidemic or successful containment), sustaining vaccination as far as possible would be the obvious choice because doing so would not substantially affect the risk of SARS-CoV-2 infection.

We assumed that the risk from COVID-19, and hence the potential disruption to health services, including routine childhood vaccination, lasts for 6 months. The main analyses consider the impact of continuation of all five immunisation clinic visits in comparison with the risk of COVID-19 disease in the vaccinated child's household as a result of attending the vaccine clinic, tracking the health benefits from immunisation among the vaccinated children until 5 years of age.

### Benefits of sustained routine childhood immunisation

We used the health impact estimates provided by Li and colleagues^3^ for vaccines against hepatitis B, *H influenzae* type b, measles, *N meningitidis* serogroup A, *S pneumoniae*, rotavirus, rubella, and yellow fever. For the health impact of vaccines against diphtheria, tetanus, and pertussis, we calculated basic estimates for the annual number of deaths averted per 1000 vaccinated children by DTP3 in Africa, on the basis of global annual DTP3 vaccine impact estimates from 1980 to 2013.^17^ We did not include polio-vaccine-preventable mortality in our estimates. Antigen-specific estimates of per-capita deaths averted by vaccination were unavailable for nine countries (Algeria, Botswana, Equatorial Guinea, Gabon, Libya, Mauritius, Namibia, Seychelles, and South Africa) and were approximated to the mean estimates of other countries with available data. Country-specific and antigen-specific levels of routine vaccination coverage were assumed to be the same in 2020 as in 2018.

The child deaths averted by routine vaccination during a 6-month suspension period of immunisation are the product of country-specific and antigen-specific estimates of per-capita deaths averted by vaccination from the time of vaccination until 5 years of age,^3^, ^17^ country-specific population estimates of the vaccinated cohort,^18^ country-specific and antigen-specific official country reported estimates of vaccination coverage,^19^ and the suspension period of immunisation.

We considered a high-impact scenario and a low-impact scenario for approximating the effects of sustaining routine childhood immunisation during the COVID-19 pandemic. In the high-impact scenario, we approximated the impact of sustaining routine childhood immunisation with use of the estimates of vaccination impact for a 6-month cohort in 2020. Hence, this scenario assumes that the suspension of immunisation will result in a cohort of unvaccinated children who have the same risk of disease as children in a completely unvaccinated population, and their vulnerability persists until they are 5 years old (ie, no catch-up campaign conducted at the end of the SARS-CoV-2 outbreak). Because of herd protection and likely catch-up activities at the end of a potential disruption of immunisation services, this high-impact scenario is likely to overestimate the negative impact of suspending immunisation services for a short period.

By contrast, in the low-impact scenario, we attempted to estimate a lower bound for the expected number of deaths due to disruptions to routine childhood immunisation services. We assumed that, in the absence of immunisation, herd immunity would protect children missing vaccination for all diseases with the exception of measles, and that vaccination through catch-up campaigns would close measles immunity gaps immediately following the 6-month COVID-19 disruption period. This scenario was implemented as illustrated by the following example. In a country with 80% routine measles vaccine coverage, the interepidemic period of measles outbreaks is about 4 years.^20^ The suspension of the routine vaccination programme for 6 months would correspond to an accumulation of susceptible individuals equivalent to 30 months in normal times, thus shrinking the interepidemic period to 2 years. In the absence of supplementary immunisation activities, this shorter period would yield a 25% chance that an outbreak would start during the 6 months of suspension. Furthermore, the physical distancing interventions in place to mitigate COVID-19 risk could decrease that outbreak probability by an additional 50%. Thereby, there is a 12·5% (25% × 50%) chance of a measles outbreak during the 6-month suspension period. In this low-impact scenario, the health impact of routine childhood immunisation includes only a 12·5% proportion of the health benefits derived from measles vaccination, while excluding the health impact of the other vaccines.^21^

Supplementary immunisation activities for 17 African countries in 2020 are either currently postponed or of unknown status and reflect a higher risk of measles outbreaks in comparison with the low-impact scenario used in this study.^22^ If routine childhood immunisation programmes are also suspended, the further decline in vaccination coverage enhances the risk of measles outbreaks in the near future.

### Excess risk of COVID-19 disease

We assume that in the coming months African countries will experience SARS-CoV-2 spread similar to that observed in non-African countries affected earlier in the pandemic, which were unable to contain the virus. We assume that the warmer climates in Africa will not notably reduce the transmissibility of SARS-CoV-2.^23^, ^24^

The risk of COVID-19 depends on exposure probability to SARS-CoV-2 and progression to disease. For this analysis, we consider only the infection-fatality risk for COVID-19 and ignore other potentially severe health outcomes. We model the additional SARS-CoV-2 exposure risk for the vaccinated child, their carer, and household members as a result of contact with the vaccinator and other community members during travel to the vaccine clinic. The simulation parameters for SARS-CoV-2 infection dynamics were based on the Reed-Frost epidemic model (appendix 1 p 2).^25^ The COVID-19 risk model is described in more detail in appendix 1 (pp 3–4). We used the country-specific household age composition to approximate the age distribution in households at risk of SARS-CoV-2 infection, given that one of the household members is a child who has been vaccinated (appendix 1 p 5).^26^ We applied age-stratified infection-fatality risk for SARS-CoV-2 using estimates obtained from reported cases and their severity in China, in combination with the proportion of asymptomatic infections estimated among international residents repatriated from China.^27^ For children (<20 years), we used the reported risk for ages 0–9 years; for adults (20–59 years), we used the reported risk for ages 30–39 years; and for older adults (>60 years), we used the reported risk for ages 60 years and older (appendix 1 p 5).

### Sensitivity analysis

We did a probabilistic sensitivity analysis by conducting 4000 simulation runs to account for the uncertainty around the parameters governing the SARS-CoV-2 infection model, as well as the reported uncertainty ranges for the infection fatality rate estimates (modelled using a γ distribution) and the vaccine-preventable mortality estimates (modelled using a log-normal distribution), and assessed their impact on our findings. We constructed a tornado diagram using a multivariate Poisson regression model fitted to the estimated posterior distribution of the benefit–risk ratio using our model input parameters as predictors, and treating total deaths averted by childhood immunisation as a single variable.

The program code and data for the benefit–risk analysis in this study are accessible online. All analyses were done using R 3.6.0.^28^ All data were from secondary sources in the public domain, and ethics approval was thereby not required.

### Role of the funding source

The funders were involved in study design, data collection, data analysis, data interpretation, writing of the report, and the decision to submit for publication. All authors had full access to all the data in the study and had final responsibility for the decision to submit for publication.

## Results

In the high-impact scenario, we estimate that the current routine childhood immunisation programme (DTP, hepatitis B, *H influenzae* type b, *S pneumoniae*, rotavirus, measles, rubella, *N meningitidis* serogroup A, and yellow fever) in Africa during a 6-month period in 2020 will prevent around 702 000 (95% uncertainty interval [UI] 635 000–782 000) deaths in children from the time of vaccination until they are 5 years old (table 1). About a third of averted deaths are attributable to measles and another third to pertussis. Immunisation during the three EPI-1 visits (three-dose vaccinations for DTP, hepatitis B, *H influenzae* type b, and *S pneumoniae*, and vaccination for rotavirus) could prevent approximately 471 000 (406 000–548 000) deaths. Immunisation during the EPI-2 visit (first dose of vaccination for measles [MCV1] and rubella [RCV1] and vaccination for *N meningitidis* serogroup A and yellow fever) is estimated to prevent around 220 000 (205 000–236 000) deaths, and immunisation during the EPI-3 visit (second dose for measles [MCV2]) to prevent 10 300 (9400–11 200) deaths among children until they are 5 years old. A third of the deaths prevented by routine childhood vaccination are predicted to be in Nigeria, Democratic Republic of the Congo, Ethiopia, and Tanzania (table 2).

**Table 1:** Vaccine antigen-specific benefits and risks of sustaining routine childhood immunisation in Africa during the COVID-19 pandemic

	**Vaccination schedule**	**Deaths averted by vaccination (95% UI)**	**Excess COVID-19 deaths (95% UI)**	**Benefit–risk ratio (95% UI)**
Diphtheria	6, 10, 14 weeks	12 944 (10 180–16 539)	5674 (846–16 830)	2 (0–7)
Tetanus	6, 10, 14 weeks	69 254 (54 268–87 343)	5674 (846–16 830)	12 (2–39)
Pertussis	6, 10, 14 weeks	271 422 (207 238–344 147)	5674 (846–16 830)	48 (8–155)
Hepatitis B	6, 10, 14 weeks	3827 (2578–5826)	5677 (846–16 837)	1 (0–2)
*Haemophilus influenzae* type b	6, 10, 14 weeks	54 840 (49 521–61 230)	5696 (849–16 896)	10 (2–30)
*Streptococcus pneumoniae*	6, 10, 14 weeks	46 494 (40 002–55 014)	5052 (752–14 979)	9 (2–29)
Rotavirus	6, 10 weeks	10 666 (9578–11 890)	2391 (364–7221)	4 (1–14)
Measles (MCV1)	9 months	194 388 (181 469–209 379)	1896 (228–5778)	103 (16–332)
Rubella (RCV1)	9 months	1147 (738–1679)	744 (85–2264)	2 (0–5)
*Neisseria meningitidis* serogroup A	9 months	460 (335–665)	280 (34–856)	2 (0–6)
Yellow fever	9 months	23 345 (17 426–30 929)	875 (100–2664)	27 (4–87)
Measles (MCV2; EPI-3)	15–18 months	10 282 (9354–11 237)	751 (81–2277)	14 (2–45)
EPI-1*	6, 10, 14 weeks	471 068 (406 088–548 290)	5696 (849–16 896)	82 (14–261)
EPI-2†	9 months	219 726 (204 572–235 744)	1896 (228–5778)	116 (18–374)
EPI‡	6, 10, 14 weeks; 9 months; 15–18 months	701 828 (635 416–782 050)	8341 (1280–25 029)	84 (14–267)

The benefit–risk ratio estimates (median estimates and 95% UIs) show the child deaths averted by sustaining routine childhood immunisation in Africa per COVID-19 death attributable to excess severe acute respiratory syndrome coronavirus 2 infections acquired through visiting routine vaccination service delivery points. Note that the vaccine-preventable death estimates are vaccine antigen-specific, whereas the excess deaths are dependent on the number of required visits. Because vaccination visits EPI-1 and EPI-2 group the delivery of several vaccines, these have a higher benefit–risk ratio than that for individual antigens. EPI=Expanded Programme on Immunization. UI=uncertainty interval.

* EPI-1 includes three-dose vaccinations for diphtheria, tetanus, and pertussis, hepatitis B, *Haemophilus influenzae* type b, and *Streptococcus pneumoniae*, and vaccination for rotavirus.

† EPI-2 includes the first dose of vaccination for measles (MCV1) and rubella (RCV1) and vaccination for *Neisseria meningitidis* serogroup A and yellow fever.

‡ EPI includes all vaccinations in EPI-1 and EPI-2, as well as the second dose for measles (MCV2; EPI-3).

**Table 2:** Benefits and risks of sustaining routine childhood immunisation at the national level

	**Deaths averted by vaccination (95% UI)**	**Excess COVID-19 deaths (95% UI)**	**Benefit–risk ratio (95% UI)**
Algeria	18 164 (11 750–28 146)	268 (29–794)	69 (10–234)
Angola	26 156 (18 377–36 792)	146 (24–434)	180 (28–598)
Benin	7285 (4817–11 246)	78 (8–228)	95 (14–311)
Botswana	989 (646–1511)	15 (2–43)	70 (11–233)
Burkina Faso	14 103 (9626–20 955)	180 (20–534)	80 (13–259)
Burundi	8640 (5946–12 784)	79 (13–238)	110 (19–367)
Cameroon	13 031 (8735–19 862)	176 (20–519)	75 (11–249)
Cape Verde	148 (85–251)	3 (0–9)	52 (6–176)
Central African Republic	2353 (1585–3513)	20 (3–60)	119 (21–392)
Chad	9016 (5998–13 984)	98 (10–288)	94 (11–310)
Comoros	420 (267–661)	7 (1–22)	58 (7–197)
Congo (Brazzaville)	3370 (2366–4919)	21 (3–63)	160 (19–515)
Côte d'Ivoire	19 401 (12 712–28 863)	194 (20–571)	102 (16–339)
Democratic Republic of the Congo	61 538 (41 399–92 956)	563 (88–1674)	111 (18–371)
Djibouti	273 (173–435)	5 (1–14)	58 (8–203)
Egypt	24 593 (11 655–48 336)	412 (54–1221)	60 (6–216)
Equatorial Guinea	360 (231–564)	4 (0–13)	83 (12–273)
Eritrea	2103 (1380–3152)	29 (4–88)	74 (9–245)
eSwatini	346 (180–603)	9 (1–28)	38 (4–136)
Ethiopia	60 854 (38 286–95 401)	866 (100–2558)	73 (10–243)
Gabon	866 (554–1360)	8 (1–25)	105 (17–358)
Gambia	2208 (1560–3107)	39 (6–117)	58 (10–189)
Ghana	18 589 (12 358–26 534)	219 (32–658)	86 (14–281)
Guinea	9307 (6339–13 372)	121 (17–362)	78 (11–255)
Guinea-Bissau	1355 (960–1912)	12 (1–36)	113 (18–379)
Kenya	20 030 (12 691–31 736)	241 (38–720)	86 (13–292)
Lesotho	829 (536–1263)	15 (2–43)	57 (8–195)
Liberia	3965 (2913–5689)	34 (4–101)	118 (17–381)
Libya	2323 (1517–3463)	34 (5–103)	70 (11–230)
Madagascar	14 293 (9228–22 263)	136 (21–405)	107 (16–359)
Malawi	8923 (5398–14 737)	131 (20–393)	69 (10–232)
Mali	13 302 (9259–18 971)	144 (17–426)	94 (14–308)
Mauritania	2720 (1905–4119)	30 (3–90)	91 (13–310)
Mauritius	260 (177–391)	4 (1–11)	71 (11–230)
Morocco	7273 (3698–13 837)	221 (26–657)	34 (4–124)
Mozambique	20 206 (13 487–30 366)	208 (33–624)	98 (14–317)
Namibia	1179 (768–1812)	19 (2–56)	63 (9–214)
Niger	21 835 (15 854–30 867)	262 (30–776)	85 (13–278)
Nigeria	89 167 (61 172–133 594)	942 (98–2773)	96 (16–316)
Rwanda	8061 (5274–12 053)	87 (14–260)	94 (15–318)
São Tomé and Príncipe	120 (72–186)	1 (0–4)	91 (12–296)
Senegal	11 306 (7856–15 866)	250 (37–758)	46 (6–154)
Seychelles	34 (23–50)	0 (0–1)	74 (10–245)
Sierra Leone	6891 (5096–9376)	86 (11–256)	81 (12–266)
Somalia	9697 (6695–14 275)	102 (11–300)	96 (16–319)
South Africa	18 844 (12 575–28 607)	310 (36–920)	62 (10–209)
South Sudan	3176 (1716–5251)	34 (5–102)	93 (11–322)
Sudan	22 338 (13 975–34 110)	334 (49–1003)	68 (10–231)
Tanzania	36 630 (23 570–56 036)	584 (87–1757)	64 (8–209)
Togo	4933 (3206–7819)	56 (6–164)	90 (13–307)
Tunisia	1854 (723–3626)	54 (8–163)	35 (3–128)
Uganda	20 906 (12 346–34 358)	246 (39–734)	87 (12–299)
Zambia	11 042 (7453–16 810)	121 (19–361)	93 (13–312)
Zimbabwe	7759 (5269–11 124)	94 (14–284)	83 (12–278)

The benefit–risk ratio estimates (median estimates and 95% UIs) show the child deaths averted by sustaining routine childhood immunisation in the African countries per COVID-19 death attributable to excess severe acute respiratory syndrome coronavirus 2 infections acquired through visiting routine vaccination service delivery points. The combined impact of routine childhood immunisation is shown, including all vaccinations in EPI-1, EPI-2, and EPI-3. EPI=Expanded Programme on Immunization. UI=uncertainty interval.

We estimate that the three immunisation visits for EPI-1 add a total of 2·4% (0·7–7·6) and each immunisation visit of EPI-2 and EPI-3 adds 0·8% (0·2–2·7) to the probability of excess SARS-CoV-2 infection in the household. As a result, continuation of routine childhood immunisation in Africa could lead to 8300 (1300–25 000) excess deaths attributable to additional SARS-CoV-2 infections associated with the immunisation visits of children. About eight (0–40) of these are expected to be among the vaccinated children, nine (0–45) among their siblings, 914 (83–2800) among their parents or adult carers, and 7300 (852–22 300) among older adults in the household.

**Figure 1:**
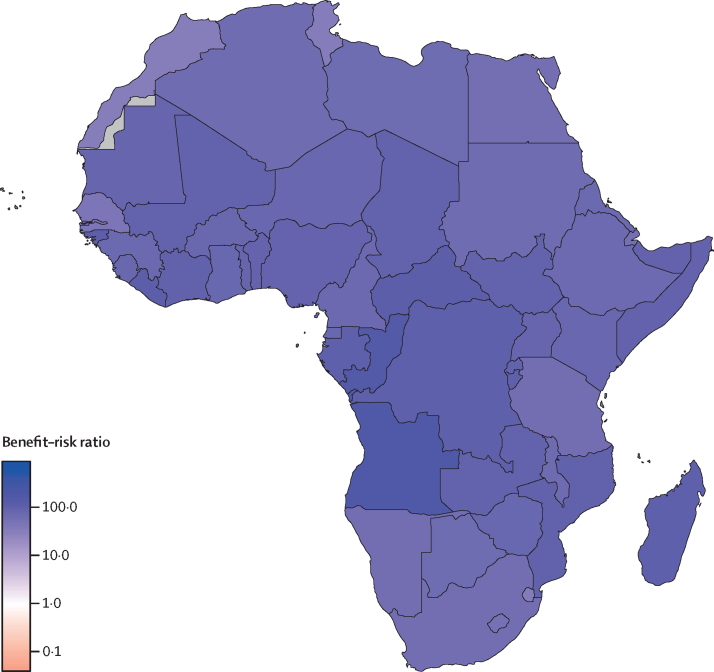
Benefit–risk ratios for sustaining routine childhood immunisation during the COVID-19 pandemic in Africa In this scenario, we assume that the suspension of immunisation will result in a cohort of unvaccinated children who have the same risk of disease as children in a completely unvaccinated population, and their vulnerability persists until they are 5 years old (ie, no catch-up campaigns). A benefit–risk ratio greater than 1 indicates in favour of sustaining the routine childhood immunisation programme. Countries shaded in grey had missing data.

For every one excess COVID-19 death attributable to additional household exposure to SARS-CoV-2 infection due to routine childhood immunisation visits in this high-impact scenario, we estimate that the routine childhood immunisation programme would prevent 84 (95% UI 14–267) deaths in children up to 5 years of age in Africa (table 1). The benefit of the three EPI-1 immunisation visits in early infancy and the visit for EPI-2 at 9 months was 82 (14–261) and 116 (18–374) deaths averted among children per excess COVID-19 death, respectively. The incremental benefit of the second dose of measles vaccination during the EPI-3 visit at 15–18 months was 14 (2–45) deaths averted among children per excess COVID-19 death. Almost 90% of the excess COVID-19 risk is due to the high fatality rate among older adults (>60 years; 96 [14–307] deaths averted per excess COVID-19 death). If only the risk to vaccinated children is considered, the benefit–risk ratio is substantially higher at 85 000 (4900–546 000) deaths averted per excess COVID-19 death (appendix 1 p 6). The benefit–risk ratio for the siblings (<20 years) of vaccinated children is 75 000 (4400–483 000), and for their parents or adult carers (20–60 years) is 769 (148–2700). Our findings were largely similar across countries (figure 1, table 2; appendix 1 pp 7–9, appendix 2). Country-specific benefit–risk ratios for EPI-1, EPI-2, and EPI-3 are presented in appendix 1 (pp 10–12). The overall benefit–risk ratio of sustaining routine childhood immunisation ranged from 34 (4–124) in Morocco to 180 (28–598) in Angola, and the number of child deaths averted through vaccination substantially exceeded the number of excess COVID-19 deaths for all 54 countries in Africa.

**Figure 2:**
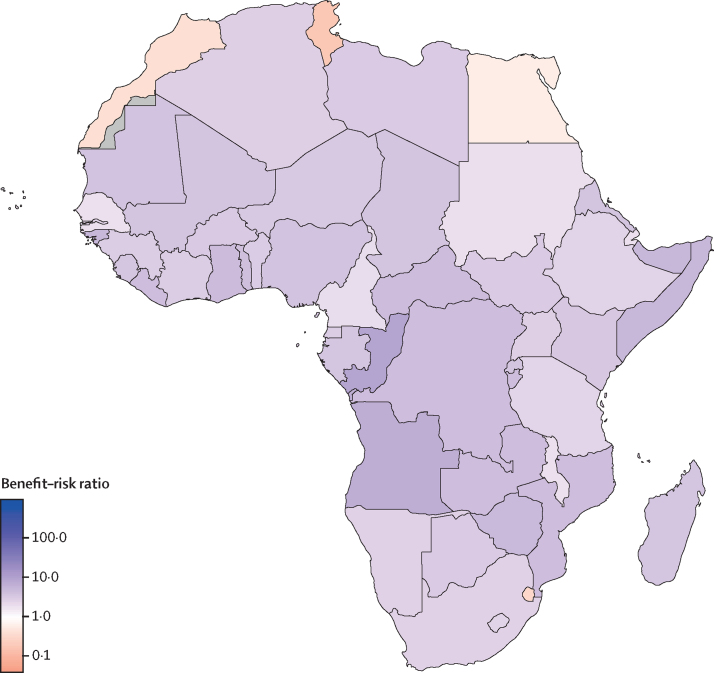
Benefit–risk ratios of sustaining routine childhood vaccination, with a minimal chance of a measles outbreak and no other vaccine-preventable outbreaks, during the COVID-19 pandemic in Africa In this scenario, we assumed that, in the absence of immunisation, herd immunity would protect children missing vaccination for all diseases except measles. We assumed that the chance of a measles outbreak during the 6-month suspension of immunisation was 12·5%, and no other vaccine-preventable disease outbreaks occurred. Countries shaded in grey had missing data.

In the low-impact scenario, which approximates the health benefits to only the child deaths averted from measles outbreaks, the benefit–risk ratio to the households of vaccinated children is 3 (0–10). If the risk to only the vaccinated children is considered, the benefit–risk ratio is 3000 (182–21 000). Even with these highly conservative assumptions, the benefit–risk ratio for most countries in Africa is greater than 1, indicating in favour of sustaining the routine childhood immunisation programme during the COVID-19 pandemic (figure 2). Tunisia, eSwatini, Morocco, and Egypt have benefit–risk ratios of less than 1 because measles vaccination impact is lower in these countries compared with other countries in Africa (appendix 2). Although the lower bounds of the uncertainty intervals of the benefit–risk ratios at the household level are less than 1 for some countries, the lower bounds of the benefit–risk ratios for the vaccinated children are greater than 1 for all countries.

**Figure 3:**
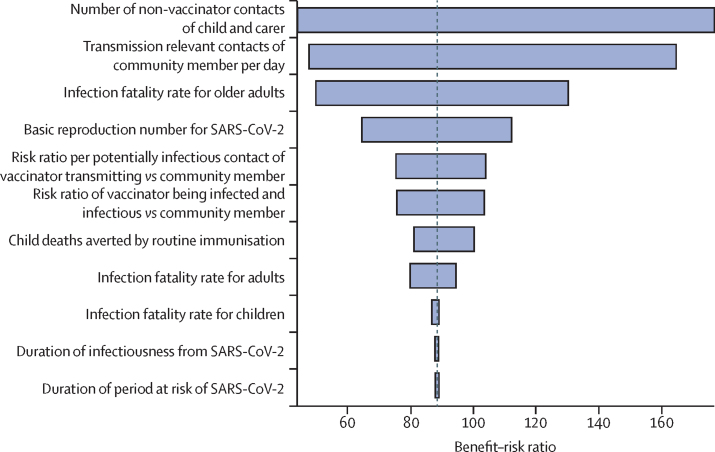
Sensitivity analysis for uncertainty in the benefit–risk ratio estimates The tornado diagram was constructed using a multivariate Poisson regression model fitted to the estimated posterior distribution of the benefit–risk ratio using our model input parameters as predictors, and treating total deaths averted by childhood immunisation as a single variable. SARS-CoV-2=severe acute respiratory syndrome coronavirus 2.

We evaluated the contribution of the uncertainty in the model parameters to the uncertainty in the benefit–risk ratio estimates (figure 3). The main factors affecting our estimates of the benefit–risk ratio were the average number of contacts of the child and their carer during a visit to the vaccine clinic, the average number of transmission-relevant contacts of a community member per day and therefore the risk for transmission given a potentially infectious contact, and the infection fatality rate for older adults (>60 years) with SARS-CoV-2 infection.

## Discussion

Our analysis suggests that the benefit of sustaining routine childhood immunisation in Africa far outweighs the excess risk of COVID-19 deaths due to the additional risk of SARS-CoV-2 infection during the child's vaccination visit, particularly for the vaccinated children. This finding reinforces the guidance issued by WHO^14^ and a statement from the Measles & Rubella Initiative^29^ which assert that routine childhood immunisation programmes should be sustained if essential health services have operational capacity of adequate human resources and vaccine supply, while maintaining physical distancing and other infection control measures to ensure the safety of communities and health workers.

We based our analyses on model-based country-specific and antigen-specific vaccine impact estimates in low-income and middle-income countries for 2020.^3^, ^17^ There is considerable uncertainty regarding the impact of suspending immunisation activities for a period of about 6 months and whether a timely and high-coverage catch-up campaign can be conducted soon after. Therefore, we presented two extreme scenarios, a high-impact scenario and a low-impact scenario, to evaluate the potential benefits of sustaining routine childhood vaccination.

In the high-impact scenario, we approximated the impact of sustained routine childhood immunisation with the estimates of vaccination impact for a 6-month cohort in 2020. This is a basic estimate of the likely impact, which, in reality, is also governed by herd protection and physical distancing. Although pathogen resurgence will happen gradually due to herd protection from the rest of the population and potentially only once physical distancing is lifted, this could be counterbalanced by unvaccinated children of this and other cohorts continuing to be at risk of disease beyond the 6-month period. In the presence of physical distancing measures, the exposure to non-coronavirus pathogens will also probably be reduced, but those who remain susceptible as a result of immunisation service suspension could get infected once physical distancing measures are relaxed. In the low-impact scenario, we approximated the impact of sustaining vaccination by the number of child deaths as a result of potential measles outbreaks during the COVID-19 risk period, assuming that catch-up campaigns will be delivered at the end of the COVID-19 risk period. We showed that, in both scenarios, continuation of routine childhood immunisation is beneficial and outweighs the excess risk of COVID-19 deaths due to the additional risk for SARS-CoV-2 infections during the immunisation visits, especially for the vaccinated children. Of note, SARS-CoV-2 infections acquired by the vaccinated children or their adult carers and parents at the vaccination clinics pose a risk primarily for the older adults in their households. This finding highlights the importance of shielding older adults to lower their risk of acquiring SARS-CoV-2 infections, while allowing children in their households to benefit from routine vaccination to lower their risk of acquiring vaccine-preventable infectious diseases.^30^

Although the young age demographic in Africa might mitigate some of the COVID-19 disease burden, infection fatality rates in Africa could be substantially higher because of the prevalence of likely risk factors, including HIV, tuberculosis, and malnutrition, and poor access to antibiotics to reduce the risk of bacterial co-infections in some parts of Africa. In the event that infection fatality rates in Africa turn out to be higher than elsewhere, then the estimated benefit–risk ratio would be reduced. However, our uncertainty analysis shows that although the uncertainty of the COVID-19 infection fatality rate is a key factor in the overall uncertainty of our estimates, even at the upper mortality bounds, sustaining routine childhood vaccination is beneficial. Furthermore, the effects of a potentially higher COVID-19 case-fatality ratio in Africa might be balanced by a higher case-fatality ratio of measles and the other vaccine-preventable diseases in times when the health-care system is stretched, treatment supplies are disrupted, and access to drugs such as vitamin A and antibiotics is reduced.

Our findings were similar across countries with respect to the benefit–risk ratios, indicating in favour of sustaining the childhood immunisation programmes during the COVID-19 pandemic in Africa. Although there will be heterogeneity in the implementation of and compliance with prevention and control measures for COVID-19 in different countries, the benefits of sustaining childhood immunisation far outweigh the risks of excess SARS-CoV-2 infections acquired during the vaccination visits, especially for the vaccinated children.

Because of high transmissibility of measles, routine childhood immunisation coverage in many countries is insufficient to prevent outbreaks. To aid routine vaccination coverage, supplementary immunisation activities are conducted regularly, and many were scheduled for 2020, at a time shortly before sufficient accumulation of susceptible children had built up to trigger measles outbreaks.^31^ Many of these supplementary immunisation activities have been postponed to reduce the risk of SARS-CoV-2 infections during mass vaccination,^14^, ^31^ further enhancing the likelihood and impact of measles outbreaks. Because they are timed at specific intervals to avoid outbreaks, our low-impact scenario is likely to underestimate the risk of an outbreak occurring due to suspension of routine childhood immunisation. Although this risk might be mitigated in part by physical distancing in response to COVID-19, the risk of outbreaks will be concentrated in the periods when interventions are gradually lifted and before a catch-up campaign can be done.

We did a probabilistic sensitivity analysis to assess the impact of parameter uncertainty on the estimated benefit–risk ratios. We found that the biggest contributors to the uncertainty around the benefit of sustaining routine childhood immunisation during the COVID-19 pandemic in Africa were the transmission probability and the number of contacts during a vaccination visit. These factors highlight the need for personal protective equipment for vaccinators, the need to implement physical distancing measures, including the avoidance of crowded waiting rooms for vaccination visits, and the importance of good hygiene practices to reduce the risk of SARS-CoV-2 acquisition and transmission at the vaccination clinics. Although the implementation of some of these infection prevention and control measures in many African countries will be challenging due to resource constraints, the risks can be minimised and the benefits can be enhanced by providing immunisation bundled with other health services, thereby reducing the number of physical touch points with the health system.

Our study has limitations and other factors must be considered during strategic decision making on sustaining routine childhood immunisation in African countries during the COVID-19 pandemic. These factors include logistical constraints of vaccine supply and delivery cold-chain problems caused by the COVID-19 pandemic, reallocation of doctors and nurses to other prioritised health services, health-care staff shortages caused by SARS-CoV-2 infections among the staff, staff shortages because of ill-health or underlying health conditions that put staff members at increased risk for severe COVID-19 disease, logistical and resource constraints for implementation of SARS-CoV-2 infection prevention and control measures at the vaccination service delivery points, and decreased demand for vaccination arising from community reluctance to visit vaccination clinics for fear of contracting SARS-CoV-2 infection or broader distrust of vaccines fuelled by COVID-19-related rumours and misinformation. The risk of SARS-CoV-2 infection for the vaccinated children and health-care staff involved in immunisation activities, as well as to their households and onward SARS-CoV-2 transmission into the wider community, should also be considered (appendix p 13).

Although we have estimated benefit–risk ratios on the basis of deaths averted, this analysis could be extended to estimate benefit–risk ratios on the basis of disability-adjusted life-years averted or quality-adjusted life-years gained. Because the deaths averted by vaccination are primarily among children younger than 5 years and deaths caused by COVID-19 are primarily among older adults, the benefit–risk ratios will be relatively higher using disability-adjusted life-years averted or quality-adjusted life-years, and will be more favourable towards sustaining routine childhood immunisation programmes during the COVID-19 pandemic in Africa.

In conclusion, routine childhood immunisation programmes should be safeguarded for continued service delivery and prioritised for the prevention of infectious diseases, as much as is logistically possible, as part of delivering essential health services during the COVID-19 pandemic in Africa.

## References

[1] Andre FE, Booy R, Bock HL (2008). Vaccination greatly reduces disease, disability, death and inequity worldwide. Bull World Health Organ.

[2] Ozawa S, Mirelman A, Stack ML, Walker DG, Levine OS (2012). Cost-effectiveness and economic benefits of vaccines in low- and middle-income countries: a systematic review. Vaccine.

[3] Li X, Mukandavire C, Cucunubá ZM (2019). Estimating the health impact of vaccination against 10 pathogens in 98 low and middle income countries from 2000 to 2030. medRxiv.

[4] Berkley S (2019). The power of vaccines and how Gavi has helped make the world healthier: 2019 Lasker-Bloomberg Public Service Award. JAMA.

[5] Piot P, Larson HJ, O'Brien KL (2019). Immunization: vital progress, unfinished agenda. Nature.

[6] WHO (April, 2020). Immunization agenda 2030: a global strategy to leave no one behind. https://www.who.int/immunization/immunization_agenda_2030/en.

[7] WHO (July, 2019). WHO-UNICEF estimates of DTP3 coverage. https://apps.who.int/immunization_monitoring/globalsummary/timeseries/tswucoveragedtp3.html.

[8] WHO (2020). Coronavirus disease (COVID-19) pandemic. https://www.who.int/emergencies/diseases/novel-coronavirus-2019.

[9] Roberton T, Carter ED, Chou VB (2020). Early estimates of the indirect effects of the COVID-19 pandemic on maternal and child mortality in low-income and middle-income countries: a modelling study. Lancet Glob Health.

[10] Clark A, Jit M, Warren-Gash C (2020). Global, regional, and national estimates of the population at increased risk of severe COVID-19 due to underlying health conditions in 2020: a modelling study. Lancet Glob Health.

[11] Huang C, Wang Y, Li X (2020). Clinical features of patients infected with 2019 novel coronavirus in Wuhan, China. Lancet.

[12] WHO (April 13, 2020). Coronavirus disease 2019 (COVID-19) situation report—84. https://www.who.int/docs/default-source/coronaviruse/situation-reports/20200413-sitrep-84-covid-19.pdf.

[13] Nelson R (2020). COVID-19 disrupts vaccine delivery. Lancet Infect Dis.

[14] WHO (March 26, 2020). Guiding principles for immunization activities during the COVID-19 pandemic. https://apps.who.int/iris/bitstream/handle/10665/331590/WHO-2019-nCoV-immunization_services-2020.1-eng.pdf.

[15] Pan American Health Organization (March 26, 2020). The immunization program in the context of the COVID-19 pandemic. https://www.paho.org/en/documents/immunization-program-context-covid-19-pandemic-march-2020.

[16] Bwaka A, Bita A, Lingani C (2019). Status of the rollout of the meningococcal serogroup A conjugate vaccine in african meningitis belt countries in 2018. J Infect Dis.

[17] Feikin D, Flannery B, Hamel M, Stack M, Hansen P, Black R, Temmerman M, Laxminarayan R, Walker N (2016). Vaccines for children in low- and middle-income countries. Disease control priorities, 3rd edn: volume 2, reproductive, maternal, newborn, and child health.

[18] UN Department of Economic and Social Affairs (2019). World population prospects 2019. https://population.un.org/wpp.

[19] WHO (Dec 10, 2019). Baccille Calmette Guérin vaccine: reported estimates of BCG coverage. https://apps.who.int/immunization_monitoring/globalsummary/timeseries/tscoveragebcg.html.

[20] WHO (May, 1999). WHO guidelines for epidemic preparedness and response to measles outbreaks. https://www.who.int/csr/resources/publications/measles/whocdscsrisr991.pdf.

[21] Sudfeld CR, Navar AM, Halsey NA (2010). Effectiveness of measles vaccination and vitamin A treatment. Int J Epidemiol.

[22] WHO (2020). Vaccine-preventable diseases: supplementary immunization activities from 2000 to 2020. https://www.who.int/immunization/monitoring_surveillance/data/Summary_Measles_SIAs.xls.

[23] O'Reilly KM, Auzenbergs M, Jafari Y, Liu Y, Flasche S, Lowe R (2020). Effective transmission across the globe: the role of climate in COVID-19 mitigation strategies. Lancet Planet Health.

[24] Martinez-Alvarez M, Jarde A, Usuf E (2020). COVID-19 pandemic in west Africa. Lancet Glob Health.

[25] Abbey H (1952). An examination of the Reed-Frost theory of epidemics. Hum Biol.

[26] UN Department of Economic and Social Affairs (2019). Household size & composition, 2019. https://population.un.org/household.

[27] Verity R, Okell LC, Dorigatti I (2020). Estimates of the severity of coronavirus disease 2019: a model-based analysis. Lancet Infect Dis.

[28] R Core Team (2019). R: a language and environment for statistical computing.

[29] Measles & Rubella Initiative (April 14, 2020). More than 117 million children at risk of missing out on measles vaccines, as COVID-19 surges. https://measlesrubellainitiative.org/measles-news/more-than-117-million-children-at-risk-of-missing-out-on-measles-vaccines-as-covid-19-surges.

[30] van Zandvoort K, Jarvis CI, Pearson C (2020). Response strategies for COVID-19 epidemics in African settings: a mathematical modelling study. medRxiv.

[31] WHO (May 22, 2020). At least 80 million children under one at risk of diseases such as diphtheria, measles and polio as COVID-19 disrupts routine vaccination efforts, warn. https://www.who.int/news-room/detail/22-05-2020-at-least-80-million-children-under-one-at-risk-of-diseases-such-as-diphtheria-measles-and-polio-as-covid-19-disrupts-routine-vaccination-efforts-warn-gavi-who-and-unicef.

