# Correction to *Lancet Glob Health* 2020; 8: e1264–72

**DOI:** 10.1016/S2214-109X(20)30419-8

**Published:** 2020-09-24

*Abbas K, Procter SR, van Zandvoort K, et al. Routine childhood immunisation during the COVID-19 pandemic in Africa: a benefit–risk analysis of health benefits versus excess risk of SARS-CoV-2 infection.* Lancet Glob Health *2020;* 8: *e1264–72.* In this Article, some of the lower bounds of uncertainty intervals for benefit–risk ratios were rounded down to 0. These have been provided to one decimal place to avoid misinterpretation. These corrections have been made as of Sept 24, 2020.

