# Supplementary material for: Routine childhood immunisation during the COVID-19 pandemic in Africa: a benefit–risk analysis of health benefits versus excess risk of SARS-CoV-2 infection
Source: Lancet Glob Health. 2020 Jul 17;8(10):e1264–72. doi: 10.1016/S2214-109X(20)30308-9 (PMC7367673; doi:10.1016/S2214-109X(20)30308-9)
Supplement: Supplementary appendix 1 [file mmc1.pdf]

THE LANCET
Global Health

Supplementary appendix 1

This appendix formed part of the original submission and has been peer reviewed. We post it as supplied by the authors.

Supplement to: Abbas K, Procter SR, van Zandvoort K, et al. Routine childhood immunisation during the COVID-19 pandemic in Africa: a benefit–risk analysis of health benefits versus excess risk of SARS-CoV-2 infection. *Lancet Glob Health* 2020; published online July 17. http://dx.doi.org/10.1016/S2214-109X(20)30308-9.

Supplementary appendix

A1. Simulation parameters for SARS-CoV-2 infection dynamics

A2. COVID-19 risk model

A3. Household structure, age composition and infection fatality risk

A4. Age and antigen specific benefit-risk ratios for Africa at the continental level

A5. Country and age specific benefit-risk ratios for Africa at the national level

A6. Benefit-risk ratio of vaccines delivered in the first, second, and third vaccination-related clinical visits

A7. Benefit-risk ratio of vaccines delivered in the fourth vaccination-related clinical visit

A8. Benefit-risk ratio of vaccines delivered in the fifth vaccination-related clinical visit

A9. Opportunity risk for vaccinated children and healthcare staff involved in immunisation activities

A10. Age and antigen specific deaths averted by vaccination, excess deaths due to COVID-19, and benefit-risk ratios for Africa at the continental level

A11. Country, age, and antigen specific deaths averted by vaccination, excess deaths due to COVID-19, and benefit-risk ratios for Africa at the national level

A12. Age and antigen specific deaths averted by measles vaccination, excess deaths due to COVID-19, and benefit-risk ratios for Africa at the continental level – Scenario of measles-only vaccination impact

A13. Country and age specific deaths averted by measles vaccination, excess deaths due to COVID-19, and benefit-risk ratios for Africa at the national level – Scenario of measles-only vaccination impact

A1. Simulation parameters for SARS-CoV-2 infection dynamics

Table A1. Parameters governing the estimation of SARS-CoV-2 infection dynamics during immunisation visits – baseline values & 95% uncertainty intervals for probabilistic sensitivity analyses.

Parameter	Description	Value	Source / calculation
v	Number of vaccine clinic visits:<br>EPI-1: 3 visits for DTP3-HepB-Hib, PCV3, RotaC	3	[1]
	EPI-2: 1 visit for MCV1, RCV1, MenA, YF<br>EPI-3: 1 visit for MCV2	1	
R_o	Basic reproduction number for SARS-CoV-2	2.5<br>(1.6 - 3.6)	[2]<br>*gamma* (mean=2.5, shape=25)
T	Duration of period at risk for SARS-CoV-2	5.5 months<br>(5.025 - 5.975)	[3]<br>*uniform* (min=5, max=6)
Θ	Proportion of SARS-CoV-2 infected population at the end of the COVID-19 risk period	60%<br>(38 - 72)	$1 - 1/R_o$
Ψ	Duration of infectiousness	7 days<br>(4 - 11)	[4]<br>*gamma* (mean=7, shape=14)
p_o	Prevalence of infectious community members on any given day	2.36%<br>(1.16 - 4.14)	$\Theta\Psi / T$
p_v	Prevalence of infectious vaccinators on any given day	5.64%<br>(1.94 - 12.9)	$\iota_1 p_o$
ι_1	Risk ratio of a vaccinator being infected and infectious versus another community member	2.5<br>(1.07 - 3.92)	assumption<br>*uniform* (min=1, max=4)
ι_2	Risk ratio per potentially infectious contact of a vaccinator transmitting versus another community member	0.62<br>(0.27 - 0.98)	assumption<br>*uniform* (min=0.25, max=1)
N	Average number of transmission relevant contacts of a community member per day	6<br>(2.2 - 9.8)	[5]<br>*uniform* (min=2, max=10)
t_o	Probability of transmission given potentially infectious contact with community members	0.063<br>(0.025 - 0.206)	$R_o / N\Psi$
t_v	Probability of transmission given potentially infectious contact with vaccinators	0.038<br>(0.011 - 0.146)	$\iota_2 t_o$
n	Number of non-vaccinator contacts of child and carer during their travel to the vaccine clinic and in the waiting room	5.5<br>(1.2 - 9.8)	assumption<br>*uniform* (min=1, max=10)
P	Probability for SARS-CoV-2 infection for the whole household of a child who gets vaccinated<br>EPI-1: 3 visits for DTP3-HepB-Hib, PCV3, RotaC	0.059<br>(0.014 - 0.210)	$P = 1 - (1 - p_v t_v)^{2v}(1 - p_o t_o)^{2vn}$
	EPI-2: 1 visit for MCV1, RCV1, MenA, YF<br>EPI-3: 1 visit for MCV2	0.020<br>(0.005 - 0.076)	
P_E	Probability for excess SARS-CoV-2 infection for the whole household of a child who gets vaccinated<br>EPI-1: 3 visits for DTP3-HepB-Hib, PCV3, RotaC	0.024<br>(0.007 - 0.076)	$P (1 - \Theta)$
	EPI-2: 1 visit for MCV1, RCV1, MenA, YF<br>EPI-3: 1 visit for MCV2	0.008<br>(0.002 - 0.027)	

A2. COVID-19 risk model

The risk of infection with SARS-CoV-2 depends on the stage of the epidemic, with relatively higher risk during the early incline phase of the epidemic and larger proportion of susceptible population and relatively lower risk during the late decline phase of the epidemic and smaller proportion of susceptible population. We refer excess risk to additional infections among households that are attributable to the vaccination visits, that these additional infections among household members would not have occurred during the course of the epidemic if not for the vaccination visits.

As a base case, we assume that through contact reducing interventions, community SARS-CoV-2 transmission will be spread over a period (T) of 5 to 6 months and the exposure risk is constant during that time due to contact-reducing interventions successfully mitigating sharp peaks in disease (Table A1) [3]. We assume that these measures will be gradually lifted and that, in the absence of vaccination visits, between $\Theta = 40\%$ and $\Theta = 70\%$ of the population will have been infected with SARS-CoV-2. This corresponds for example to the herd immunity threshold for a basic reproduction number (R_o) of between 1.6 and 3.6 assuming that everyone who is infected develops full immunity. Partial immunity following infection combined with a reduction in effective reproduction number following physical distancing measures would also achieve a final epidemic size of around this level. It follows from above that between 30% and 60% of the population would not have become infected with SARS-CoV-2 independent of whether or not the infants in their households had attended routine childhood vaccination. Furthermore, if after 6 months 60% of the population was infected then, assuming a duration of infectiousness (Ψ) of one week [4] and a reasonably flat epidemic curve, then on any given day about $p_o \sim 2\%$ of the population would be infected and potentially transmitting. In comparison to community members, we assume that vaccinators are at higher risk of being infected (between 1 and 4 times, $p_v = \iota_1 p_o$) because of their higher frequency of exposure to other people, but at lower risk of onward transmission (between 0.25 and 1 times, $t_v = \iota_2 t_o$) because most of their contacts with vaccinees are brief, and they have enhanced risk awareness and use corresponding protective measures including basic respiratory hygiene and personal protective equipment as available. Also, we assume that an infant child and the parent or adult carer each have between 1 and 10 ($n = U(1, 10)$) potentially infectious contacts during their travel to the vaccine clinic and in the waiting room.

For each of the potentially infectious contacts by the child and parent with community members, there is a probability of transmission ($t_o = R_o / N\Psi$), which for example corresponds to ($t_o \sim 6\%$) probability of a transmission event occurring for ($R_o = 2.5$) secondary infections for someone with 6 contacts per day during their infectious period of 7 days (i.e., a community member) or 21 potentially infectious contacts per day but who self isolates on symptom onset that occurred 2 days into their infectious period (i.e., a vaccinator).

Both the vaccinated child and the parent or caregiver, will be at additional risk of exposure during travel to the vaccine clinic, while waiting at the vaccine clinic and during vaccination. In addition, we

assume that if either of them gets infected they will infect all other household members, owing to the high secondary attack rates observed for family gatherings [6]. We ignore any additional secondary infections outside the household, which are likely to be minimal due to physical distancing measures.

Based of the Reed-Frost epidemic model [7], the probability (P) for a SARS-CoV-2 infection for the whole household of a child who gets vaccinated is calculated as one minus the probability of either the infant or the mother not being infected by either the vaccinator or anyone else on any of the vaccination visits: $P = 1 - (1 - p_v t_v)^{2\nu}(1 - p_o t_o)^{2\nu n}$, with v the number of vaccine clinic visits. Hence, the probability for such infection to be in excess of SARS-CoV-2 infections that would have occurred otherwise is $P_E = P (1 - \Theta)$.

We assume that during the 6 months of SARS-CoV-2 transmission, all children who get one dose of DTP will also get the other two doses. However, children receiving their measles containing vaccines will only get one dose during that time window because the two doses are given more than six months apart. The number of children who would normally get DTP during the considered time frame is approximated by half of the under one-year old population. Similarly, the number of children who will get either the first or the second measles-containing vaccine dose is half of the under 1-year old children or half of the children aged 12-23 months respectively.

A3. Household structure, age composition and infection fatality risk

We use the country-specific household age composition to approximate the age distribution in households at risk of SARS-CoV-2 infection given that one of the household members is a child who has been vaccinated [8]. First, we estimate the number of siblings of an infant from the average number of household members aged less than 20 in households with at least one member aged less than 20. The number of siblings is adjusted to account for the effect of birth order by assuming that on average the infant would be the mid-born child. Secondly, we assume the average household will have two adults (parents or caregivers). Thirdly we assume that a proportion of households with vaccinated children will also have 2 older adults aged over 60 years. We estimate this proportion using the percentage of households that have both members aged less than 20 years and over 60 years old.

To estimate the number of COVID-19 deaths in infected households, we applied age-stratified infection fatality risk for SARS-CoV-2 using estimates obtained from reported cases and their severity in China in combination with the proportion of asymptomatic infections estimated among international residents repatriated from China [9]. For children, we used the reported risks for ages 0-9 years, for adults the risk for ages 30-39 years, and for older adults the risk for ages 60 years and above. To account for uncertainty in these estimates we used gamma distributions fitted to the reported uncertainty in these risks.

Table A3. Infection fatality risk parameters used in sensitivity analysis to estimate the number of COVID-19 deaths amongst infected households, based on model-based analysis estimates of the severity of COVID-19 [9].

Infection fatality rate	Probability distribution	Value
Children (aged < 20 years)	*gamma* (shape=1.716739, rate=867.9138)	0.00161% (0.00019 - 0.00586)
Adults (aged 20 - 59 years)	*gamma* (shape=8.550388, rate=97.24091)	0.08464% (0.03926 - 0.15629)
Older adults (aged ≥ 60 years)	*gamma* (shape=12.18207, rate=3.607364)	3.28379% (1.73737 - 5.53980)

A4. Age and antigen specific benefit-risk ratios for Africa at the continental level

Table A4. Age-and antigen-specific benefit-risk ratios for childhood vaccination during the COVID-19 pandemic in Africa at the continental level. The benefit-risk ratio estimates (central estimates and uncertainty intervals) show the child deaths averted by continuing the routine childhood immunisation programmes per excess COVID-19 death caused by SARS-CoV-2 infections acquired in the vaccination service delivery points in Africa. The routine childhood vaccines considered are 3-dose DTP3, HepB3, Hib3, PCV3 for children at 6, 10 and 14 weeks, 2-dose RotaC for children at 6 and 10 weeks, 1-dose MCV1, RCV1, MenA, YFV for children at 9 months, and 1-dose MCV2 for children at 15-18 months of age. Benefit-risk ratio above 1 indicates in favour of sustaining the routine childhood immunisation programme during the COVID-19 pandemic. The health benefits are accrued by the vaccinated children while the excess COVID-19 risk is disaggregated across the different age groups in the household.

Vaccine	Benefit-risk ratios				
	Household	Vaccinated children	Siblings (< 20 years of age)	Adults (20-60 years of age)	Older adults (> 60 years of age)
Diphtheria (DTP3)	2 (0-7)	2,293 (139-14,926)	2,014 (122-13,107)	21 (4-72)	3 (0-9)
HepB3	1 (0-2)	686 (33-4,688)	602 (29-4,116)	6 (1-22)	1 (0-3)
Hib3	10 (2-30)	9,796 (724-61,874)	8,593 (635-54,275)	88 (14-299)	11 (2-35)
MCV1	103 (16-332)	103,318 (6,602-675,385)	90,700 (5,796-592,902)	934 (147-3,218)	117 (17-384)
MCV2	14 (2-45)	13,620 (708-87,942)	13,107 (682-84,629)	123 (17-425)	16 (2-51)
PCV3	9 (2-29)	9,489 (706-61,053)	8,210 (611-52,826)	86 (14-294)	10 (2-34)
Pertussis (DTP3)	48 (8-155)	47,980 (2,858-320,376)	42,132 (2,510-281,330)	441 (70-1,509)	54 (10-179)
RCV1	2 (0-5)	1,601 (82-10,670)	1,417 (72-9,441)	14 (2-51)	2 (0-6)
RotaC	4 (1-14)	4,732 (258-30,782)	4,241 (232-27,583)	43 (7-145)	5 (1-16)
Tetanus (DTP3)	12 (2-39)	12,436 (728-80,478)	10,920 (639-70,670)	111 (14-385)	14 (2-46)
YFV	27 (4-87)	27,616 (1,747-184,358)	21,749 (1,376-145,190)	248 (32-849)	31 (4-102)
MenA	2 (0-6)	1,750 (94-11,681)	1,355 (73-9,041)	16 (2-56)	2 (0-6)
DTP3, HepB3, Hib3, PCV3, RotaC	82 (14-261)	83,947 (5,017-542,744)	73,638 (4,401-476,092)	762 (107-2,595)	94 (16-303)
MCV1, RCV1, MenA, YFV	116 (18-374)	116,813 (6,428-759,071)	102,547 (5,643-666,367)	1,055 (142-3,615)	132 (20-431)
DTP3, HepB3, Hib3, PCV3, RotaC, MCV1, RCV1, MenA, YFV, MCV2	84 (14-267)	84,674 (4,948-546,123)	74,892 (4,378-483,020)	769 (148-2,679)	96 (14-307)

A5. Country and age specific benefit-risk ratios for Africa at the national level

Table A5. Country and age specific benefit-risk ratios of vaccines delivered in the five vaccination-related clinical visits (3-dose DTP3, HepB3, Hib3, PCV3; 2-dose RotaC; 1-dose MCV1, RCV1, MenA, YFV, MCV2) during the COVID-19 pandemic in Africa at the country level. The benefit-risk ratio estimates (central estimates and uncertainty intervals) show the child deaths averted by continuing the routine childhood immunisation programmes per excess COVID-19 death caused by SARS-CoV-2 infections acquired in the vaccination service delivery points in Africa. The routine childhood vaccines considered are 3-dose DTP3, HepB3, Hib3, PCV3 for children at 6, 10 and 14 weeks, 2-dose RotaC for children at 6 and 10 weeks, 1-dose MCV1, RCV1, MenA, YFV for children at 9 months, and 1-dose MCV2 for children at 15-18 months of age. Benefit-risk ratio above 1 indicates in favour of sustaining the routine childhood immunisation programme during the COVID-19 pandemic. The health benefits are accrued by the vaccinated children while the excess COVID-19 risk is disaggregated across the different age groups in the household.

Country	Benefit-risk ratios				
	Household	Vaccinated children	Siblings (< 20 years of age)	Adults (20-60 years of age)	Older adults (> 60 years of age)
Angola	180 (27-598)	109,538 (7,310-737,985)	87,995 (5,872-592,844)	980 (160-3,539)	227 (33-768)
Burundi	110 (19-367)	87,595 (4,436-601,184)	78,952 (3,998-541,869)	788 (110-2,836)	131 (16-438)
Benin	95 (14-311)	102,469 (7,929-691,613)	81,029 (6,270-546,905)	936 (159-3,329)	107 (13-360)
Burkina Faso	80 (13-259)	87,158 (4,459-587,520)	60,767 (3,109-409,621)	794 (105-2,760)	90 (15-298)
Botswana	70 (11-233)	78,334 (4,606-512,881)	92,693 (5,450-606,895)	695 (94-2,436)	79 (11-268)
Central African Republic	119 (21-392)	96,886 (4,313-642,618)	75,191 (3,347-498,720)	874 (117-3,022)	139 (22-470)
Côte d'Ivoire	101 (16-339)	110,308 (4,615-755,640)	93,351 (3,906-639,479)	999 (119-3,545)	114 (17-390)
Cameroon	75 (11-249)	91,086 (4,892-598,300)	70,761 (3,800-464,792)	820 (103-2,897)	84 (14-287)
Congo - Kinshasa	111 (18-371)	91,517 (5,667-634,575)	68,305 (4,230-473,625)	823 (95-2,924)	130 (20-445)
Congo - Brazzaville	160 (19-515)	111,185 (7,334-785,032)	112,994 (7,453-797,803)	1,006 (164-3,597)	192 (31-659)
Comoros	58 (7-197)	84,958 (4,504-575,765)	73,037 (3,872-494,975)	772 (113-2,742)	63 (9-219)
Cape Verde	52 (6-176)	56,845 (3,012-384,901)	48,598 (2,575-329,064)	514 (68-1,917)	58 (7-202)

Djibouti	58 (8-203)	63,130 (3,235-431,468)	53,972 (2,766-368,875)	581 (59-2,109)	66 (9-233)
Algeria	69 (10-234)	74,399 (4,421-511,411)	63,606 (3,780-437,221)	679 (108-2,475)	78 (10-272)
Egypt	60 (6-216)	40,251 (2,074-302,147)	55,285 (2,848-414,995)	370 (39-1,458)	73 (7-272)
Eritrea	74 (9-245)	80,033 (4,051-526,855)	68,423 (3,463-450,424)	720 (103-2,558)	83 (9-282)
Ethiopia	73 (10-243)	86,041 (5,101-592,442)	84,960 (5,037-584,994)	794 (101-2,790)	80 (10-274)
Gabon	105 (17-358)	91,578 (4,602-617,477)	84,382 (4,240-568,958)	816 (95-3,046)	122 (19-424)
Ghana	86 (14-281)	87,038 (4,608-590,327)	103,839 (5,498-704,283)	784 (119-2,766)	98 (15-323)
Guinea	78 (11-255)	128,947 (6,320-855,348)	88,565 (4,341-587,484)	1,161 (121-4,055)	84 (11-279)
Gambia	58 (9-189)	106,616 (7,317-718,292)	49,744 (3,414-335,133)	959 (161-3,361)	62 (9-204)
Guinea-Bissau	113 (18-379)	121,631 (8,076-809,856)	103,986 (6,904-692,371)	1,107 (146-3,922)	128 (19-441)
Equatorial Guinea	83 (12-273)	90,947 (3,556-610,982)	77,754 (3,040-522,348)	812 (132-2,944)	93 (13-319)
Kenya	86 (13-292)	70,406 (3,349-498,310)	81,892 (3,895-579,604)	647 (62-2,322)	99 (14-347)
Liberia	118 (17-381)	125,265 (8,995-816,096)	110,302 (7,921-718,618)	1,114 (136-3,934)	133 (22-445)
Libya	70 (11-230)	75,379 (4,094-517,031)	64,444 (3,500-442,026)	684 (108-2,411)	79 (11-264)
Lesotho	57 (8-195)	80,512 (5,119-565,273)	118,291 (7,522-830,517)	730 (83-2,655)	63 (9-216)
Morocco	34 (3-124)	43,385 (2,097-317,753)	47,309 (2,287-346,492)	395 (35-1,505)	38 (3-140)
Madagascar	107 (16-359)	85,342 (5,320-590,034)	75,798 (4,725-524,052)	775 (109-2,773)	126 (18-437)
Mali	94 (15-308)	111,820 (6,796-741,419)	81,387 (4,946-539,632)	1,012 (116-3,503)	105 (14-348)
Mozambique	98 (14-317)	77,341 (4,595-510,862)	76,661 (4,555-506,369)	704 (105-2,490)	116 (14-387)
Mauritania	91 (13-310)	100,042 (6,578-682,233)	85,529 (5,623-583,262)	902 (138-3,236)	102 (14-355)
Mauritius	71 (11-230)	76,946 (3,535-495,123)	65,783 (3,023-423,296)	686 (97-2,453)	79 (12-267)
Malawi	69 (9-232)	62,572 (3,514-434,494)	63,688 (3,577-442,248)	565 (61-2,107)	80 (10-274)
Namibia	63 (9-214)	82,182 (5,366-542,897)	81,326 (5,310-537,241)	744 (75-2,626)	70 (8-237)

Niger	85 (13-278)	100,340 (6,153-675,769)	67,251 (4,124-452,920)	904 (98-3,104)	95 (15-317)
Nigeria	96 (16-316)	98,154 (5,286-662,519)	82,605 (4,448-557,570)	889 (137-3,095)	109 (15-367)
Rwanda	94 (15-318)	77,057 (4,725-516,657)	90,199 (5,531-604,775)	693 (97-2,480)	110 (15-385)
Sudan	68 (10-231)	70,708 (4,187-473,187)	56,043 (3,318-375,045)	643 (104-2,395)	77 (10-267)
Senegal	46 (6-154)	99,845 (5,242-690,829)	45,351 (2,381-313,781)	912 (111-3,105)	49 (7-165)
Sierra Leone	81 (12-266)	121,448 (5,889-812,623)	94,585 (4,586-632,885)	1,100 (144-3,738)	89 (12-295)
Somalia	97 (16-319)	106,604 (5,416-715,223)	91,139 (4,630-611,466)	954 (118-3,365)	109 (14-365)
South Sudan	93 (11-322)	82,765 (4,355-555,572)	58,938 (3,101-395,633)	746 (115-2,810)	109 (12-381)
São Tomé and Príncipe	91 (12-296)	74,944 (4,055-508,305)	82,903 (4,486-562,283)	694 (80-2,395)	107 (13-357)
Swaziland	38 (4-136)	49,292 (2,433-349,688)	38,228 (1,887-271,198)	457 (55-1,682)	42 (6-153)
Seychelles	74 (10-245)	80,446 (4,452-559,539)	68,776 (3,806-478,367)	726 (88-2,556)	84 (13-285)
Chad	93 (11-310)	89,228 (4,713-573,267)	56,793 (3,000-364,881)	803 (102-2,786)	108 (17-367)
Togo	90 (13-307)	102,242 (5,844-693,935)	93,667 (5,354-635,735)	912 (120-3,340)	101 (13-346)
Tunisia	35 (3-128)	37,911 (1,427-278,478)	32,411 (1,220-238,079)	349 (35-1,313)	40 (4-147)
Tanzania	64 (8-209)	69,202 (4,159-458,084)	61,021 (3,667-403,929)	627 (98-2,228)	72 (10-241)
Uganda	87 (12-299)	75,924 (3,431-532,919)	65,470 (2,958-459,538)	692 (90-2,498)	101 (13-351)
South Africa	62 (10-209)	75,957 (4,232-537,132)	113,320 (6,314-801,349)	682 (91-2,445)	69 (10-233)
Zambia	93 (13-312)	78,942 (4,123-525,938)	62,676 (3,273-417,567)	709 (99-2,539)	109 (14-374)
Zimbabwe	83 (12-278)	87,567 (4,369-596,551)	101,826 (5,081-693,691)	783 (116-2,786)	95 (13-324)

A6. Benefit-risk ratio of vaccines delivered in the first, second, and third vaccination-related clinical visits

Figure A6. Benefit-risk ratio of vaccines delivered in the first, second, and third vaccination-related clinical visits (3-dose DTP3, HepB3, Hib3, PCV3; 2-dose RotaC) for children at 6, 10, 14 weeks of age during the COVID-19 pandemic in Africa. The central estimates for benefit-risk ratio at the household level show the child deaths averted by continuing the routine childhood immunisation programmes (3-dose DTP3, HepB3, Hib3, PCV3 for children at 6, 10 and 14 weeks of age and 2-dose RotaC for children at 6 and 10 weeks of age) per excess COVID-19 death caused by SARS-CoV-2 infections acquired in the vaccination service delivery points. Benefit-risk ratio above 1 indicates in favour of sustaining the routine childhood immunisation during the COVID-19 pandemic.

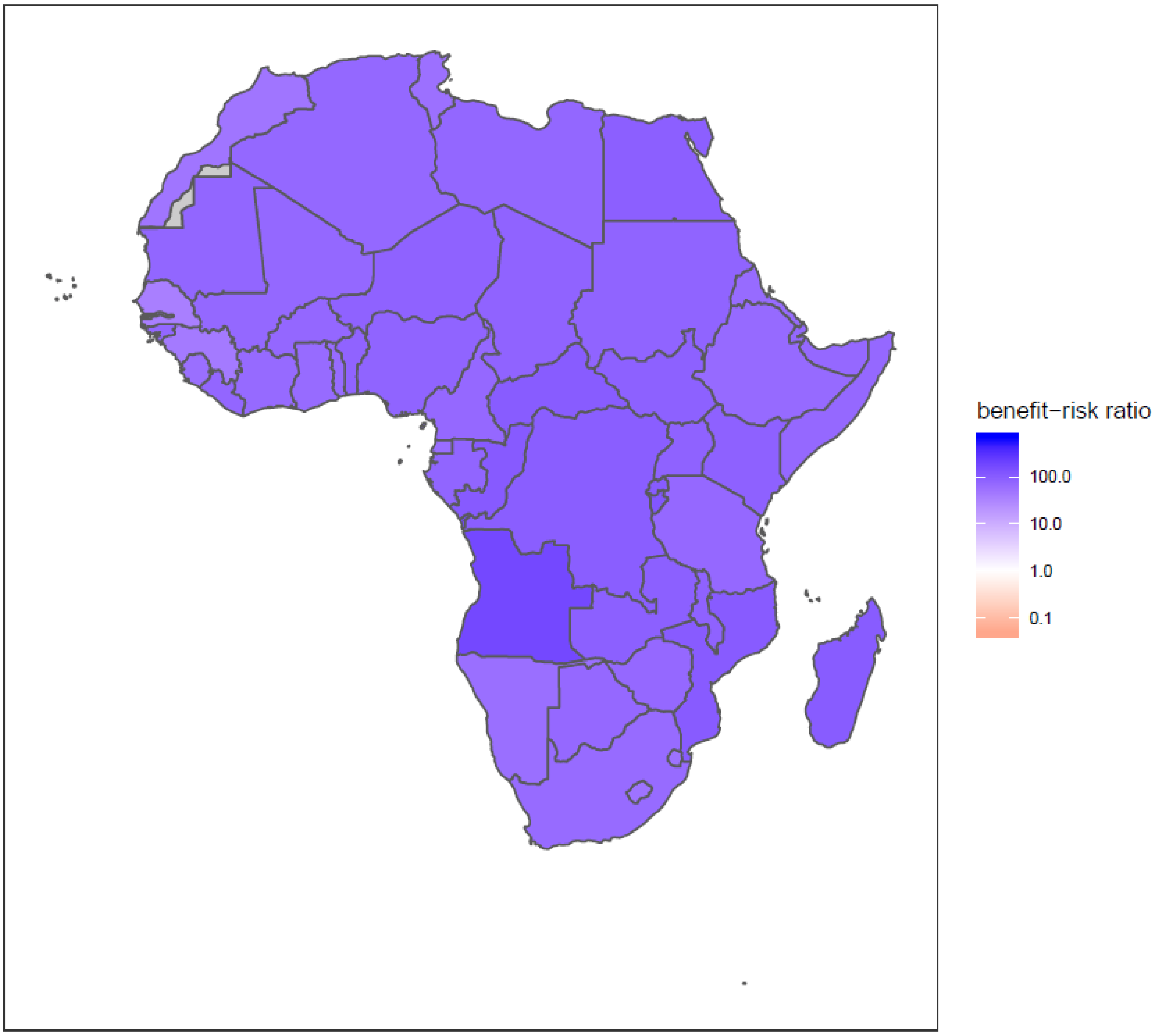

A7. Benefit-risk ratio of vaccines delivered in the fourth vaccination-related clinical visit

Figure A7. Benefit-risk ratio of vaccines delivered in the fourth vaccination-related clinical visit (1-dose MCV1, RCV1, MenA, YFV) for children at 9-months of age during the COVID-19 pandemic in Africa. The central estimates for benefit-risk ratio at the household level show the child deaths averted by continuing the routine childhood immunisation programmes (1-dose MCV1, RCV1, MenA, YFV for 9-month-old children) per excess COVID-19 death caused by SARS-CoV-2 infections acquired in the vaccination service delivery points. Benefit-risk ratio above 1 indicates in favour of sustaining the routine childhood immunisation during the COVID-19 pandemic.

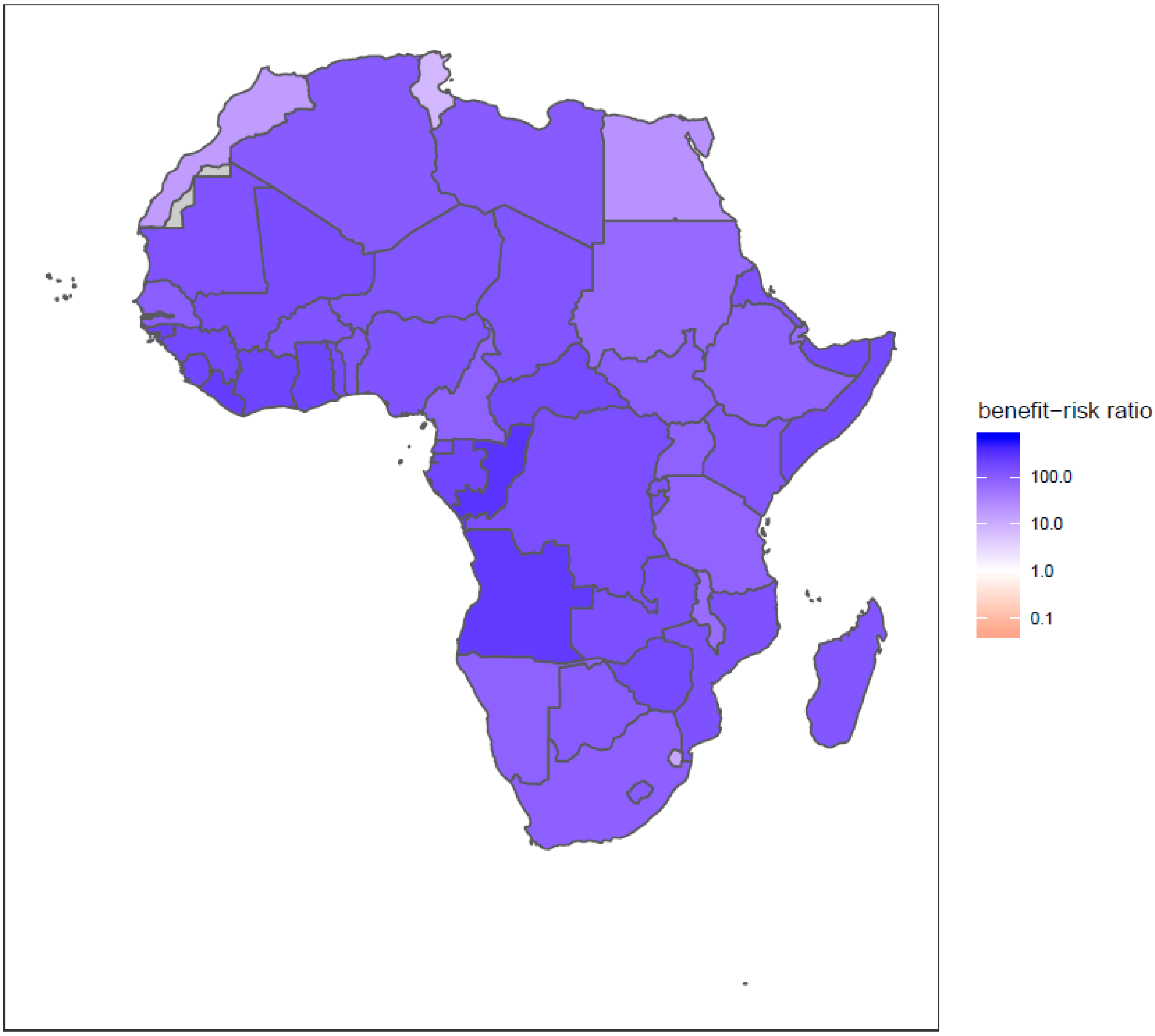

A8. Benefit-risk ratio of vaccines delivered in the fifth vaccination-related clinical visit

Figure A8. Benefit-risk ratio of vaccines delivered in the fifth vaccination-related clinical visit (1-dose MCV2) for children at 15-18 months of age during the COVID-19 pandemic in Africa. The central estimates for benefit-risk ratio at the household level show the child deaths averted by continuing the routine childhood immunisation programmes (1-dose MCV2 for children aged 15-18 months) per excess COVID-19 death caused by SARS-CoV-2 infections acquired in the vaccination service delivery points. Benefit-risk ratio above 1 indicates in favour of sustaining the routine childhood immunisation during the COVID-19 pandemic. Grey shading indicates null MCV2 coverage.

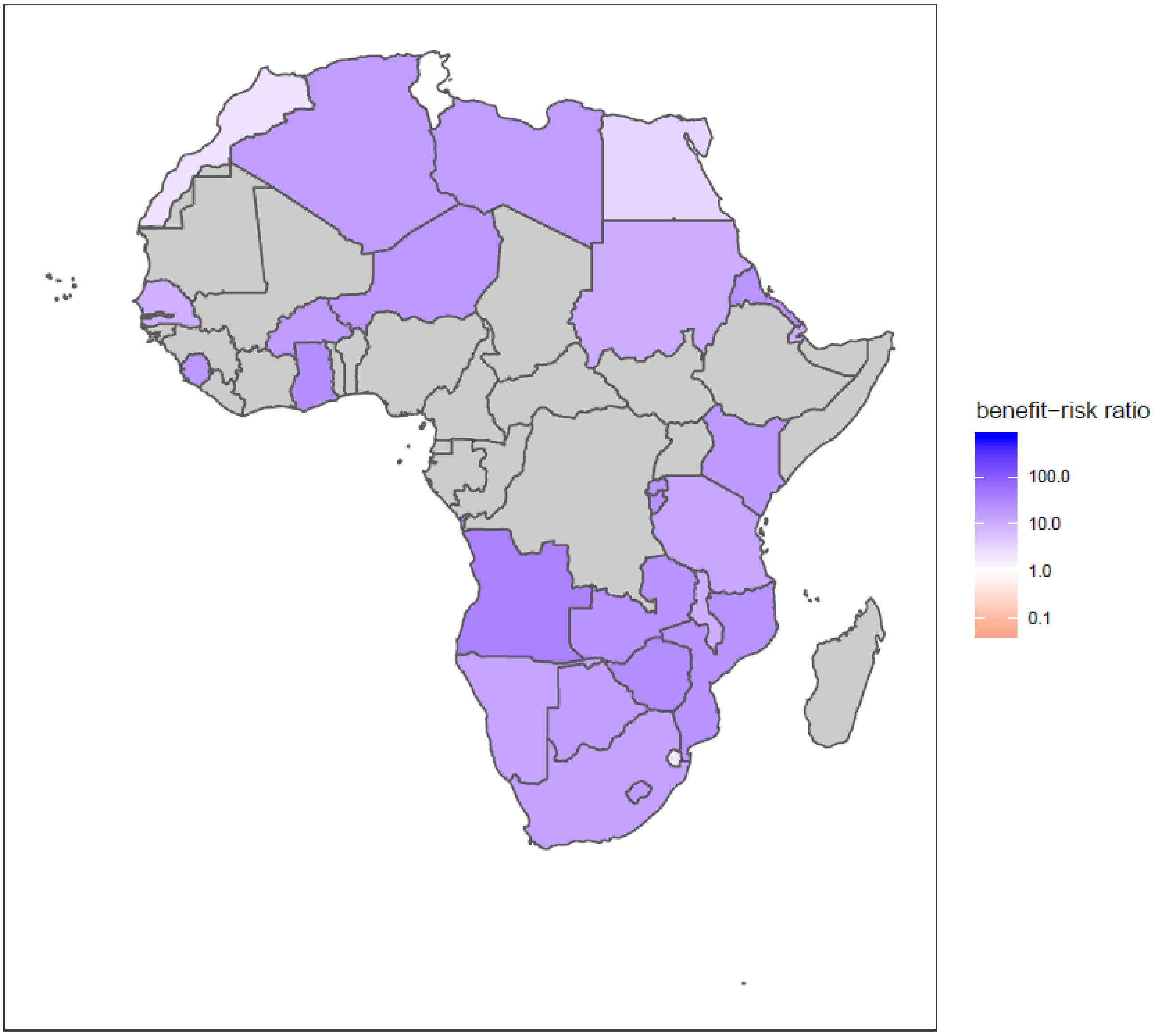

A9. Opportunity risk for vaccinated children and healthcare staff involved in immunisation activities

The opportunity risk of SARS-CoV-2 infection for the vaccinated children and healthcare staff involved in immunisation activities as well as to their households and onward SARS-CoV-2 transmission into the wider community should be included in the decision-making process to sustain routine childhood immunisation.

First, we need to know the opportunity risk of SARS-CoV-2 infection for the healthcare staff. Similar to the concept of opportunity cost, what is the risk of SARS-CoV-2 infection to the healthcare staff engaged in alternative healthcare activities if not involved in immunisation activities? If the opportunity risk of alternative healthcare activities is lower than being involved in immunisation activities, then reallocation of healthcare staff from immunisation to alternative healthcare activities is a better risk-avoidance strategy. On the other hand, if the opportunity risk of alternative healthcare activities is higher than being involved in immunisation activities, then healthcare staff face relatively lower risk in continuing to provide the immunisation services, thereby posing relatively lower risk to their households and SARS-CoV-2 transmission into the wider community.

Second, we need to know the opportunity risk of SARS-CoV-2 infection to the vaccinated children. If the alternative activity that the children and their carers would be involved in had a higher risk of SARS-CoV-2 infection in comparison to the risk involved with the immunisation visits, then it is beneficial for the children and their carers to undertake the immunisation visits for the children to get vaccinated and thereby posing relatively lower risk to their households and SARS-CoV-2 transmission into the wider community.

Irrespective of the opportunity risk of SARS-CoV-2 infection for the healthcare staff providing immunisation services during the COVID-19 pandemic, to ensure their safety, health care practices will need to be adapted to minimise the risk of SARS-CoV-2 acquisition and transmission at vaccination clinics. This includes physical distancing measures, personal protective equipment, and good hygiene practices for infection control at the vaccination clinics.

A10. Age and antigen specific deaths averted by vaccination, excess deaths due to COVID-19, and benefit-risk ratios for Africa at the continental level

Age and antigen specific deaths averted by vaccination, excess deaths due to COVID-19, and benefit-risk ratios (central estimates and uncertainty intervals) for routine childhood vaccination are included in the dataset. The routine childhood vaccines considered are 3-dose DTP3, HepB3, Hib3, PCV3 for children at 6, 10 and 14 weeks, 2-dose RotaC for children at 6 and 10 weeks, 1-dose MCV1, RCV1, MenA, YFV for children at 9 months, and 1-dose MCV2 for children at 15-18 months of age. Note that the risk is disaggregated across the different age groups in the household.

See supplementary appendix 2 (spreadsheet) for the dataset.

A11. Country, age, and antigen specific deaths averted by vaccination, excess deaths due to COVID-19, and benefit-risk ratios for Africa at the national level

Country, age and antigen specific deaths averted by vaccination, excess deaths due to COVID-19, and benefit-risk ratios (central estimates and uncertainty intervals) for routine childhood vaccination are included in the dataset. The routine childhood vaccines considered are 3-dose DTP3, HepB3, Hib3, PCV3 for children at 6, 10 and 14 weeks, 2-dose RotaC for children at 6 and 10 weeks, 1-dose MCV1, RCV1, MenA, YFV for children at 9 months, and 1-dose MCV2 for children at 15-18 months of age. Note that the risk is disaggregated across the different age groups in the household.

See supplementary appendix 2 (spreadsheet) for the dataset.

A12. Age and antigen specific deaths averted by measles vaccination, excess deaths due to COVID-19, and benefit-risk ratios for Africa at the continental level – Scenario of measles-only vaccination impact

Age specific deaths averted by measles vaccination, excess deaths due to COVID-19, and benefit-risk ratios (central estimates and uncertainty intervals) for childhood vaccination (measles-only vaccination impact) are included in the dataset. Note that the risk is disaggregated across the different age groups in the household.

See supplementary appendix 2 (spreadsheet) for the dataset.

A13. Country and age specific deaths averted by measles vaccination, excess deaths due to COVID-19, and benefit-risk ratios for Africa at the national level – Scenario of measles-only vaccination impact

Country and age specific deaths averted by measles vaccination, excess deaths due to COVID-19, and benefit-risk ratios (central estimates and uncertainty intervals) for childhood vaccination (measles-only vaccination impact) are included in the dataset. Note that the risk is disaggregated across the different age groups in the household.

See supplementary appendix 2 (spreadsheet) for the dataset.

Bibliography (for appendix)

1. WHO. WHO recommendations for routine immunization - summary tables [Internet]. 2019 [cited 1 Apr 2020]. Available: https://www.who.int/immunization/policy/immunization_tables/en/
2. Kucharski AJ, Russell TW, Diamond C, Liu Y, Edmunds J, Funk S, et al. Early dynamics of transmission and control of COVID-19: a mathematical modelling study. Lancet Infect Dis. 2020;20: 553–558. doi:10.1016/S1473-3099(20)30144-4
3. van Zandvoort K, Jarvis CI, Pearson C, Davies NG, CMMID COVID-19 working group, Russell TW, et al. Response strategies for COVID-19 epidemics in African settings: a mathematical modelling study. medRxiv. 2020; doi:10.1101/2020.04.27.20081711
4. Woelfel R, Corman VM, Guggemos W, Seilmaier M, Zange S, Mueller MA, et al. Clinical presentation and virological assessment of hospitalized cases of coronavirus disease 2019 in a travel-associated transmission cluster. medRxiv. 2020; doi:10.1101/2020.03.05.20030502
5. Prem K, Cook AR, Jit M. Projecting social contact matrices in 152 countries using contact surveys and demographic data. PLoS Comput Biol. 2017;13: e1005697. doi:10.1371/journal.pcbi.1005697
6. Liu Y, Eggo RM, Kucharski AJ. Secondary attack rate and superspreading events for SARS-CoV-2. Lancet. 2020;395: e47. doi:10.1016/S0140-6736(20)30462-1
7. Abbey H. An examination of the Reed-Frost theory of epidemics. Hum Biol. 1952;24: 201–233.
8. United Nations, Department of Economic and Social Affairs, Population Division. Database on Household Size and Composition 2019 [Internet]. 2019 [cited 1 Apr 2020]. Available: https://population.un.org/household
9. Verity R, Okell LC, Dorigatti I, Winskill P, Whittaker C, Imai N, et al. Estimates of the severity of coronavirus disease 2019: a model-based analysis. Lancet Infect Dis. 2020; doi:10.1016/S1473-3099(20)30243-7
